# Coordinated Two-Node Blockade of NF-κB and TGF-β/Smad Signaling Attenuates the Foreign Body Response to Prevent Capsular Contracture

**DOI:** 10.3390/biomedicines14071586

**Published:** 2026-07-15

**Authors:** Xiaofei Tong, Meina Sun, Xin Gan, Xin Chen, Shiyang Liu, Ieong Sun, Lin Zhang, Weihong Zheng

**Affiliations:** 1 Department of Thyroid and Breast Surgery, Tongji Hospital, Tongji Medical College, Huazhong University of Science and Technology, Wuhan 430030, China; 2 Department of Orthopedics, Tongji Hospital, Tongji Medical College, Huazhong University of Science and Technology, Wuhan 430030, China; 2025tj0043@hust.edu.cn (X.G.);

**Keywords:** emodin, liposome, capsular contracture, foreign body response, NF-κB, TGF-β/Smad

## Abstract

**Background**: Capsular contracture is driven by self-amplifying foreign body response (FBR) where inflammatory and fibrotic signals from fibroblasts and macrophages reinforce each other. We hypothesized that cogradient simultaneous blockade of NF-κB as an inflammatory node and TGF-β/Smad as fibrotic node could attenuate the FBR. Emodin has dual inhibitory activity but suffers from poor delivery. **Methods**: Emodin liposomes (Emo-Lip) were characterized and tested on TGF-β1-stimulated NIH/3T3 fibroblasts and LPS-stimulated RAW264.7 macrophages. In a rat silicone implant model, periprosthetic injections were given for four weeks. Fibrous capsule formation was evaluated by histology, immunofluorescence, and FAPI-PET/CT. Transcriptomic analyses were performed to verify and predict relevant pathways. **Results**: Emo-Lip had uniform size and high encapsulation efficiency. In vitro, Emo-Lip inhibited fibroblast migration, ROS production, myofibroblast differentiation (α-SMA^+^) as well as *Ctgf* expression, while suppressing M1 polarization and reduced IL-12/IL-6 secretion in macrophages. In vivo, Emo-Lip reduced capsule thickness, collagen area, and α-SMA/Col I expression, comparable to dexamethasone. Transcriptomics showed coordinated downregulation of inflammatory/fibrotic genes, and Western blot confirmed suppressed phosphorylation of Smad3. **Conclusions**: Coordinated two-node blockade of NF-κB and TGF-β/Smad by liposomal emodin reprograms the FBR and effectively prevents capsular contracture in rats, offering a translational strategy for implant-associated fibrosis.

## 1. Introduction

While a non-degradable implant enters the body, the immune system faces a fundamental paradox. The foreign object is too large to be engulfed and cleared, yet it cannot be left unattended. The organism compromises by walling it off with fibrous tissue [1,2]. In evolutionary terms, this constitutes sound defensive logic. When that same ancient strategy plays out in the setting of breast implant reconstruction, however, defense becomes pathology. Unchecked fibrous encapsulation leads to capsular contracture, implant deformation, pain, and eventually surgical failure [3,4,5]. For decades, materials scientists have tried to render implant surfaces more inert in hopes of evading immune surveillance, yet the incidence of capsular contracture has barely declined [6]. This impasse reveals a conceptual limitation. The crux of the foreign body response may not be how invisible a material is, but rather how the host immune system reads the signals of non-self [7,8,9]. That insight has prompted a strategic pivot, away from escaping immune recognition and toward rewriting the rules of the immune response. Conventional strategies try to make implant surfaces invisible by minimizing non-self cues, yet they overlook the fact that the host immune system actively interprets signals of non-self [10,11]. An alternative approach that actively instructs immune cell signaling pathways may therefore align more closely with the biological reality of host-material interactions [12,13,14].

Macrophages and fibroblasts are the principal actors in this pathological dialogue [15,16]. Macrophages sense danger signals on the implant surface via pattern recognition receptors, polarize toward an M1 phenotype, and secrete large amounts of pro-inflammatory mediators such as interleukin-12 (IL-12) and interleukin-6 (IL-6) [17,18,19]. These signals create a pro-fibrotic microenvironment that drives fibroblast transdifferentiation into myofibroblasts, a process marked by expression of α-smooth muscle actin (α-SMA) and excessive deposition of collagen I (COL 1) [20]. Progressive collagen accumulation eventually encases the implant in a fibrous capsule. Critically, inflammation and fibrosis engage in a self-amplifying positive feedback loop [7,21]. Inflammation promotes fibrosis, and the fibrotic milieu amplifies inflammatory signaling. Blocking only one arm of this circuit is of limited value, as the other arm can sustain the pathological axis through crosstalk [22,23]. NF-κB serves as the core transcriptional node that drives macrophage M1 polarization and the release of pro-inflammatory cytokines [24,25,26], whereas the TGF-β/Smad axis is the primary pathway that drives fibroblast activation and collagen deposition [27,28]. Because these two pathways engage in a positive feedback loop within the foreign body response, simultaneous inhibition of both nodes represents a rational strategy to dismantle this self-amplifying circuit [29,30].

A logical therapeutic strategy is to intercept both the inflammatory sensing node and the fibrotic execution node simultaneously [31]. Emodin, a natural *anthraquinone*, is pharmacologically well suited for this task. It inhibits the nuclear factor-κB (NF-κB) pathway to block M1 polarization and interferes with transforming growth factor-β (TGF-β)/Smad signaling to suppress fibroblast activation and collagen synthesis [32,33,34]. These two activities have been demonstrated independently in separate systems, but they have rarely been brought together and exploited synergistically within a single disease framework. The real bottleneck has been pharmacokinetics [35]. Emodin’s rigid polycyclic core gives it extremely low aqueous solubility and rapid systemic clearance, making it difficult to maintain an effective drug concentration locally at the implant [36,37]. A molecule with ideal dual-target activity thus remains unable to reach its intended site of action.

Liposomal delivery offers an engineering solution to bridge this gap [38]. A phospholipid bilayer encapsulates emodin within a hydrophobic core, imparting colloidal dispersibility in aqueous media [38]. The submicron particle size permits passive accumulation via the enhanced vascular permeability characteristic of inflamed tissue, while gradual bilayer degradation provides sustained release [39,40]. Through this three-tier mechanism, emodin is converted from a rapidly cleared short-lived molecule into a sustained-release modulator that persists at the implant site [41]. Upon local injection, the nanosystem establishes a therapeutic microenvironment at the implant-host interface. It suppresses inflammatory sensing in macrophages upstream and blocks the fibrotic execution program in fibroblasts downstream [7,14]. Coordinated two-node blockade represents a departure from immune evasion: rather than making the implant invisible, it actively steers the immune response away from a fibrotic outcome.

We therefore constructed a liposome-encapsulated emodin nanoplatform and systematically tested the core strategy of cooperative two-node blockade. We assessed its independent regulatory capacity in fibroblast activation and macrophage M1 polarization models. In a rat silicone implant model, we evaluated its ability to suppress fibrous capsule formation. Transcriptomic and molecular analyses then uncovered the molecular basis for its coordinated regulation of the NF-κB and TGF-β/Smad pathways. This work provides a locally applied therapeutic strategy with translational potential for implant-associated capsular contracture and serves as a proof of principle for synergistic two-node attenuation.

## 2. Materials and Methods

### 2.1. Materials

Emodin standard (purity ≥ 98%, MedChemExpress, Monmouth Junction, NJ, USA); soybean lecithin, cholesterol, chloroform, methanol, pentobarbital sodium (Aladdin, Shanghai, China); PBS, 4% paraformaldehyde, H&E and Masson’s trichrome staining kits, GAPDH and β-actin antibodies (Servicebio, Wuhan, China); DMEM high glucose, FBS, penicillin-streptomycin (100×), trypsin-EDTA (Wuhan Pricella, Wuhan, China); TGF-β1 (Biosharp, Hefei, China); CCK-8 kit, Calcein-AM, propidium iodide (Beyotime, Shanghai, China); DCFH-DA kit (Beijing Solarbio, Beijing, China); 8 μm Transwell chambers (Corning, New York, NY, USA); primary antibodies against α-SMA, Vimentin, p-Smad3, Smad3, Collagen I, CD86, F4/80, p-p65, and p65 (Abcam, Cambridge, UK); Alexa Fluor 488/594-conjugated secondary antibodies (ABclonal, Wuhan, China); IL-6 and IL-12 ELISA kits (Shanghai Yaji, Shanghai, China); LPS (MedChemExpress); silicone discs (medical grade, 1 cm diameter and 3 mm thickness, ethylene oxide sterilized); dexamethasone injection (Sinopharm, Beijing, China); TRIzol, reverse transcriptase (Thermo Fisher Scientific, Waltham, MA, USA); reverse transcription and library construction kits (Yeasen, Shanghai, China); random hexamer primers (Sangon Biotech, Shanghai, China). All reagents were of analytical grade.

Key instruments: rotary evaporator (Zhengzhou Greatwall, Zhengzhou, China); ultrasonic cell disruptor (Ningbo Scientz, Ningbo, China); DLS analyzer (LB-550, HORIBA, Kyoto, Japan); TEM (HITACHI, Tokyo, Japan); HPLC system (Wuhan Biobomei, Wuhan, China); Zeta potential analyzer (BeNano 180 Zeta, Bettersize, Dandong, China); high-speed centrifuge (Eppendorf, Hamburg, Germany); cell incubator, NanoDrop 2000 (Thermo Fisher Scientific); inverted microscope (OLYMPUS, Tokyo, Japan); microplate reader (Molecular Devices, Sunnyvale, CA, USA); confocal microscope (Leica Biosystems, Wetzlar, Germany); flow cytometer (BD FACSCanto II, Franklin Lakes, NJ, USA); tissue processor, embedding station, microtome, slide dryer, fully automated slide scanner (Pannoramic 250 FLASH III, 3DHISTECH, Budapest, Hungary); Agilent 2100 Bioanalyzer (Agilent Technologies, Santa Clara, CA, USA); Illumina NovaSeq 6000 platform (Illumina: San Diego, CA, USA).

Detailed experimental procedures are provided in the Supplementary Information.

### 2.2. Synthesis of Emo-Lip

Emodin liposomes (Emo-Lip) were prepared using the thin-film dispersion method. Briefly, emodin was dissolved in a chloroform/methanol mixed solvent (3:1, *v*/*v*), to which soybean lecithin and cholesterol were added as lipid matrix materials. Three formulations with mass ratios of emodin to total lipid of 1:20 (E1), 1:10 (E2), and 1:5 (E3) were thoroughly mixed and transferred to a round-bottom flask. Organic solvents were removed by rotary evaporation under reduced pressure in a 50 °C water bath to form a uniform pale-yellow lipid film, followed by vacuum drying to further eliminate residual solvents. The film was then dispersed in 15 mL PBS (pH 7.4) and hydrated with constant shaking at 37 °C for 4 h to obtain a crude multilamellar liposome suspension. After probe sonication for 5 min, the suspension was centrifuged at 12,000 rpm at 4 °C for 30 min, and the pellet was resuspended in PBS for subsequent experiments.

### 2.3. Characterization of Emo-Lip

Particle size, size distribution, and PDI were measured by DLS (LB-550, HORIBA) at 25 °C after 20-fold dilution with PBS. Zeta potential was recorded on a BeNano 180 Zeta (Bettersize). Morphology was examined by TEM (HITACHI, 120 kV) with negative staining. Colloidal stability in PBS (pH 7.4) at 37 °C was monitored by DLS over 14 days. Encapsulation efficiency was determined by HPLC after methanol disruption of pelleted liposomes. For uptake studies, PKH67-labeled Emo-Lip was incubated with NIH/3T3 cells for 12 or 24 h, and uptake was quantified by flow cytometry (FITC channel). In parallel, fixed cells were stained with DAPI and phalloidin and imaged by confocal microscopy (Leica).

### 2.4. NIH/3T3 Fibroblast In Vitro Fibrosis Model Including Culture, Viability Assay and Functional Assays

NIH/3T3 cells were cultured in DMEM with 10% FBS and stimulated with TGF-β1 (10 ng/mL). Groups: Control, TGF-β1, TGF-β1 + Emo-Sol (20 μM), TGF-β1 + Emo-Lip (20 μM emodin equivalent). Cell viability (CCK-8 at 6 h, 12 h and 24 h), live/dead staining (Calcein-AM/PI), Transwell migration (8 μm pores, 24 h, crystal violet staining), wound healing (scratch assay after 12 h and 24 h), and intracellular ROS (DCFH-DA probe, fluorescence microscopy) were evaluated by standard protocols. For qPCR analysis of Ctgf, total RNA was extracted from treated NIH/3T3 cells, reverse-transcribed, and amplified using SYBR Green with primers.

### 2.5. RAW264.7 Macrophage M1 Polarization Model and Inflammation Assessment

RAW264.7 cells were stimulated with LPS (100 ng/mL, 24 h). Groups: Control, LPS, LPS + Emo-Sol (20 μM), LPS + Emo-Lip (20 μM emodin equivalent). Cell viability was measured by CCK-8 assay at 12, 24, and 36 h. M1 polarization was assessed by flow cytometry (CD86/CD11b co-staining) and analyzed with FlowJo. Inflammatory cytokines in culture supernatants were quantified by ELISA (IL-6, IL-12).

### 2.6. Immunofluorescence Staining

NIH/3T3 cells and RAW264.7 macrophages were fixed (4% paraformaldehyde), permeabilized (0.1% Triton X-100), blocked (1% BSA), and incubated with primary antibodies overnight at 4 °C. NIH/3T3: anti-α-SMA (1:200) and anti-Vimentin (1:200); RAW264.7: anti-F4/80 (1:200) and anti-CD86 (1:200). Alexa Fluor-conjugated secondary antibodies were applied, nuclei were counterstained with DAPI, and images were acquired by confocal microscopy. The α-SMA^+^/Vimentin^+^ ratio and CD86^+^/F4/80^+^ ratio fluorescence intensity were quantified.

### 2.7. Western Blot Analysis

Total protein was extracted from cells or fibrous capsule tissues using RIPA buffer (with protease/phosphatase inhibitors), quantified by BCA assay, and 30–50 μg per lane was resolved by SDS-PAGE and transferred to PVDF membranes. After blocking, blots were probed with primary antibodies overnight at 4 °C. For NIH/3T3 cells: p-Smad2/3, Collagen I, and GAPDH; for RAW264.7 cells: p-p65, p65, and GAPDH; for in vivo fibrous capsule tissue: p-p65, p65, p-Smad3, Smad3, α-SMA, and GAPDH. HRP-conjugated secondary antibodies were applied, signals were developed by ECL, and grayscale analysis was performed with ImageJ (Fiji, version 2.14.0).

### 2.8. Rat Silicone Implantation Fibrous Capsule Model and In Vivo Experiment

Male SD rats (6–8 weeks, *n* = 24) were randomly divided into four groups (*n* = 6): Control (saline), Emo-Sol (20 μM, 1 mL), Emo-Lip (20 μM emodin equivalent, 1 mL), and DEX (2 mg/mL, 1 mL). Under pentobarbital anesthesia, silicone discs were implanted subcutaneously on the dorsum. Drugs were injected periprosthetically every other day for 4 weeks. All animal procedures were approved by the IACUC of Huazhong University of Science and Technology (No. 5294) at 2025.11.18.

### 2.9. FAPI-PET/CT In Vivo Imaging

Four weeks after implantation, FAPI tracer was administered via intravenous injection, and whole-body PET/CT scanning was performed. The mean standardized uptake value (SUV) of the periprosthetic region was calculated to assess the degree of fibroblast activation.

### 2.10. Tissue Harvesting, Histopathological Staining, Immunofluorescence Staining, and Safety Evaluation

At the experimental endpoint, rats were euthanized with an overdose of sodium pentobarbital, and the implants along with the surrounding fibrous capsule tissues were carefully harvested and fixed in 4% paraformaldehyde for 48 h and then sectioned (5 μm). H&E and Masson’s trichrome staining were performed for capsule thickness measurement and collagen quantification. Immunofluorescence staining for α-SMA and Collagen I was performed as described above. Major organs (liver, spleen, kidney) were harvested and processed for H&E staining to evaluate systemic toxicity.

### 2.11. Transcriptome Sequencing and Bioinformatics Analysis

Total RNA was extracted from fibrous capsule tissues (Control and Emo-Lip groups, *n* = 3) using TRIzol. After quality control (NanoDrop 2000, Agilent 2100 Bioanalyzer, RIN ≥ 8.5), mRNA was enriched, fragmented, and reverse-transcribed. Libraries were constructed and sequenced on an Illumina NovaSeq 6000 platform. DEGs were identified with DESeq2 (|log_2_FC| > 1, adjusted *p* < 0.05). Visualization was performed with ggplot2 and pheatmap. GO and KEGG enrichment analyses were conducted with clusterProfiler. The RNA-seq datasets supporting the conclusions of this article are available in the NCBI SRA repository under BioProject ID PRJNA1467595.

### 2.12. Statistical Analysis

All experiments were independently repeated three or more times. Data are presented as the mean ± standard deviation (s.d.). Comparisons among multiple groups were performed using one-way analysis of variance (one-way ANOVA), followed by the least significant difference (LSD) test for post hoc pairwise comparisons. *p* < 0.05 was considered statistically significant (* *p* < 0.05, ** *p* < 0.01, *** *p* < 0.001). Statistical analyses and graphing were performed using GraphPad Prism 8.0 software.

## 3. Results

### 3.1. Preparation and Characterization of Emodin Liposomes

**Figure 1:**
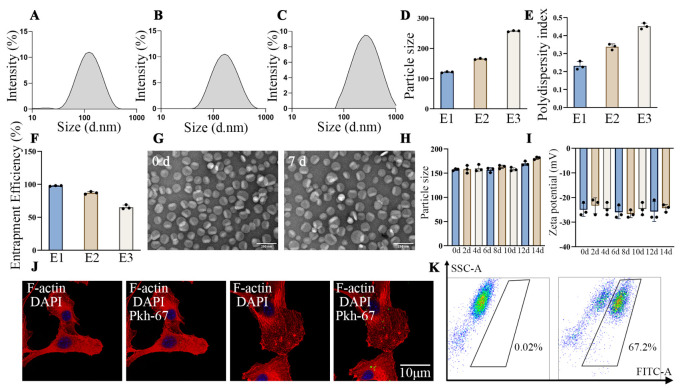
Preparation and characterization of emodin-loaded liposomes (Emo-Lip). (**A**–**C**) Size distribution profiles of Emo-Lip prepared at E1, E2, and E3 ratios, respectively, measured by dynamic light scattering (DLS). (**D**) Quantitative comparison of mean particle size among different formulation ratios. (**E**) Polydispersity index (PDI) of emodin liposomes at different formulation ratios. (**F**) Encapsulation efficiency (EE%) of Emo-Lip at different formulation ratios determined by HPLC. (**G**) Representative transmission electron microscopy (TEM) images of Emo-Lip at Day 0 and after storage at 4 °C for 7 days. Scale bar = 250 nm. (**H**) Colloidal stability of Emo-Lip stored at 4 °C over 14 days, as monitored by changes in mean particle size. (**I**) Zeta potential stability of Emo-Lip over 14 days of storage at 4 °C. (**J**) Representative confocal images of cellular uptake of PKH67-labeled Emo-Lip (green) by NIH/3T3 fibroblasts after 24 h of incubation. Nuclei were stained with DAPI (blue), and cytoskeleton was stained with phalloidin (red). Scale bar = 50 μm. (**K**) Representative flow cytometry histograms showing FITC fluorescence intensity in cells after incubation with fluorescently labeled Emo-Lip. All data are presented as mean ± s.d. *n* = 3.

Three emodin liposome formulations with different lipid and drug ratios (E1 for 1:20, E2 for 1:10, and E3 for 1:5) were prepared by thin-film dispersion and characterized. Dynamic light scattering revealed that all three formulations exhibited unimodal and narrow size distributions, as reflected by a low polydispersity index, indicating uniform particles. The mean hydrodynamic diameters were 125.3 ± 5.1 nm (E1), 168.2 ± 9.5 nm (E2), and 251.8 ± 13.8 nm (E3), with corresponding polydispersity index values of 0.22 ± 0.02, 0.33 ± 0.03, and 0.45 ± 0.04, respectively (Figure 1A–E). The E1 formulation yielded the smallest particle size and the narrowest size distribution, and was therefore selected for subsequent experiments. Encapsulation efficiency determined by HPLC was 98.5 ± 1.2% (E1), 86.3 ± 2.5% (E2), and 66.2 ± 3.8% (E3), confirming that the E1 ratio provided the most efficient drug loading (Figure 1F). Transmission electron microscopy (TEM) showed that the E1 formulation (Emo-Lip) consisted of spherical or near-spherical vesicles with sharp contours, uniform size, and good dispersion at Day 0. After storage at 4 °C for 7 days, TEM images remained virtually indistinguishable from those of the fresh preparation, with intact membrane structures and no evidence of aggregation or fusion (Figure 1G). Colloidal stability was further evaluated over 14 days at 4 °C. The mean particle size remained stable while the zeta potential showed only a minor decline, values that remain sufficiently negative to maintain electrostatic repulsion and prevent aggregation (Figure 1H,I). Cellular uptake was examined using PKH67-labeled Emo-Lip incubated with NIH/3T3 fibroblasts. Confocal microscopy showed intracellular green fluorescence after 24 h of incubation, indicating uptake of PKH67-labeled Emo-Lip by NIH/3T3 fibroblasts (Figure 1J). Flow cytometry plots demonstrated a clear shift in fluorescence intensity in the FITC channel at 24 h compared with 0 h, further confirming cellular internalization of the liposomes (Figure 1K).

### 3.2. Emo-Lip Inhibits TGF-β1-Induced Activation of NIH/3T3 Fibroblasts

**Figure 2:**
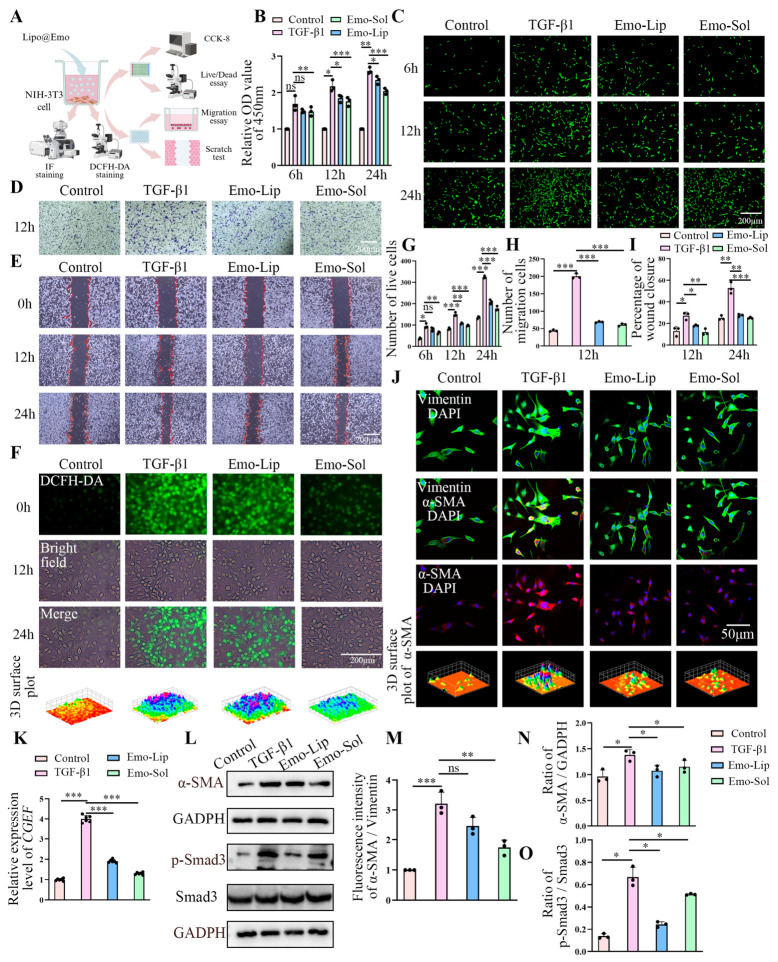
Emo-Lip inhibits TGF-β1-induced activation of NIH/3T3 fibroblasts. (**A**) Schematic illustration of the in vitro experimental design for fibroblast activation studies. (**B**) Cell viability assessed by CCK-8 assay at 6 h, 12 h, and 24 h. (**C**) Representative live/dead staining images. Live cells were stained with Calcein-AM (green) and dead cells with propidium iodide (PI, red). Scale bar = 200 μm. (**D**) Representative images of Transwell migration assay at 12 h. Migrated cells were stained with crystal violet (purple). Scale bar = 200 μm. (**E**) Representative images of wound healing assay at 0, 12, and 24 h after scratching. Scale bar = 200 μm. (**F**) Representative fluorescence images of intracellular reactive oxygen species (ROS) levels detected by DCFH-DA probe (green) at 0 h, 12 h, and 24 h, with corresponding bright-field images, merged images, and 3D surface plots. Scale bar = 200 μm. (**G**) Quantitative analysis of live cell numbers from live/dead staining. (**H**) Quantitative analysis of migrated cell numbers from Transwell assay. (**I**) Quantitative analysis of wound closure rate at 12 h from scratch assay. (**J**) Representative immunofluorescence images of α-SMA (red) and Vimentin (green) co-staining. Nuclei were counterstained with DAPI (blue). Scale bar = 50 μm. (**K**) Relative mRNA expression of *Ctgf* detected by qPCR. (**L**) Western blot analysis of α-SMA, p-Smad3 and Smad3 expression in NIH/3T3 cells. GAPDH was used as the loading control. (**M**) Quantitative analysis of α-SMA^+^/Vimentin^+^ fibroblast ratio. (**N**) Densitometric quantification of α-SMA relative to GAPDH. (**O**) Quantitative analysis of p-Smad3/Smad3 fibroblast ratio. All data are presented as mean ± s.d. * *p* < 0.05, ** *p* < 0.01, *** *p* < 0.001 versus TGF-β1 group. ns, not significant.

**Figure 3:**
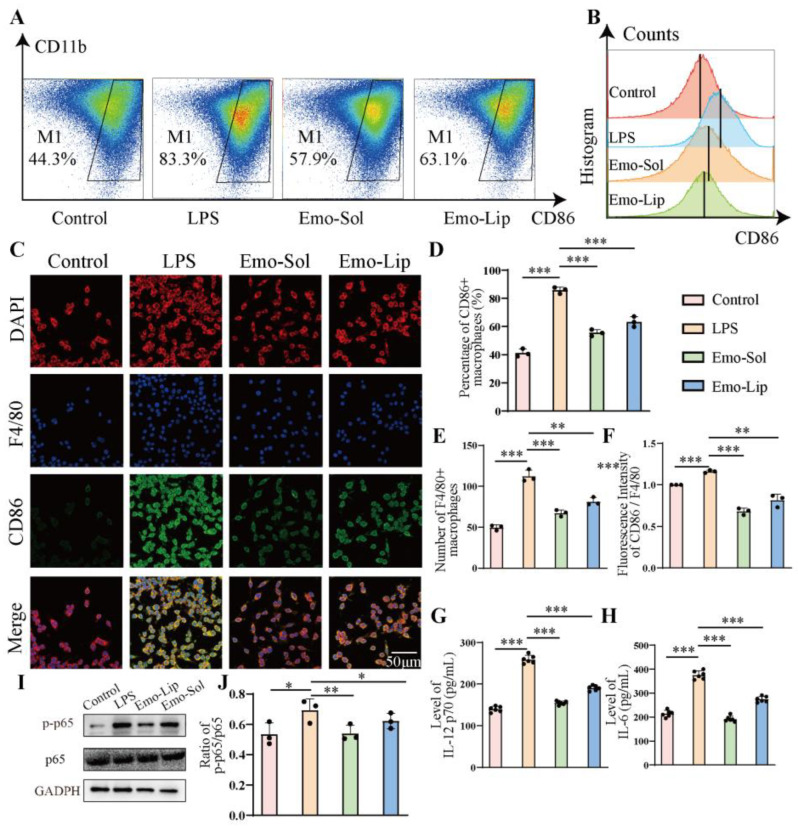
Emo-Lip suppresses LPS-induced M1 polarization in RAW264.7 macrophages. (**A**) Representative flow cytometry dot plots of CD86 and CD11b staining after 24 h treatment with LPS (100 ng/mL) alone, LPS + free emodin (Emo-Sol, 20 μM), or LPS + Emo-Lip (equivalent concentration 20 μM). (**B**) Flow cytometry histograms showing CD86 fluorescence intensity distribution across treatment groups. The line marks the peak CD86 intensity for the respective group. (**C**) Representative immunofluorescence images of F4/80 (red) and CD86 (green) co-staining. Nuclei were counterstained with DAPI (blue). Scale bar = 50 μm. (**D**) Quantitative analysis of the percentage of CD86^+^ macrophages from flow cytometry. (**E**) Quantitative analysis of F4/80^+^ cell numbers from immunofluorescence images. (**F**) Quantitative analysis of CD86^+^/F4/80^+^ fluorescence intensity ratio. Secretion levels of IL-6 (**G**) and IL-12 (**H**) in cell culture supernatants measured by ELISA. (**I**) Western blot analysis of p-p65 and total p65 protein expression levels. (**J**) Densitometric quantification of p-p65/p65 ratio. All data are presented as mean ± s.d. * *p* < 0.05, ** *p* < 0.01, *** *p* < 0.001 versus LPS group. ns, not significant.

To evaluate the effect of Emo-Lip on fibroblast activation, we established an in vitro fibrosis model using TGF-β1-stimulated NIH/3T3 fibroblasts (Figure 2A). CCK-8 assay was performed to assess cell viability, showing a modest increase compared with the untreated control after 24 h of TGF-β1 stimulation. Both Emo-Lip and free emodin slightly reduced viability, but neither treatment caused substantial viability loss (Figure 2B). Live/Dead staining confirmed this benign profile. In all treatment groups, the vast majority of cells displayed green fluorescence with only scattered red PI-positive dead cells detectable, indicating no detectable cytotoxicity at the concentrations tested. Quantitative analysis of live cell numbers revealed a statistically significant increase in the proliferation rate during TGF-β1 stimulation and both the Emo-Lip and Emo-Sol groups can inhibit its influence (Figure 2C,G). Cell migration was evaluated by Transwell assay and scratch wound healing assay. TGF-β1 stimulation markedly increased the number of cells migrating through the membrane, and this effect was significantly attenuated by both Emo-Lip and Emo-Sol treatment (Figure 2D,H). Consistently, TGF-β1-treated cells exhibited accelerated wound closure at 12 h and 24 h compared with the control group, whereas both emodin formulations slowed the closure rate (Figure 3E,I). Intracellular reactive oxygen species (ROS) levels measured using the DCFH-DA probe showed that TGF-β1 stimulation also led to a pronounced increase in intracellular ROS fluorescence intensity compared with the control group. Both Emo-Lip and Emo-Sol treatment significantly reduced ROS levels, with quantitative analysis showing that the relative fluorescence intensity of single cells in both treatment groups was significantly lower than that in the TGF-β1 group (Figure 2F).

Myofibroblast transdifferentiation was assessed by α-SMA and Vimentin co-staining, with Vimentin (green) labeling the total fibroblast population and α-SMA (red) serving as an activation marker. The α-SMA^+^/Vimentin^+^ ratio was used to quantify fibroblast activation. Untreated cells showed few α-SMA^+^ fibroblasts. Upon TGF-β1 stimulation, the proportion of α-SMA^+^/Vimentin^+^ cells increased sharply, indicating robust myofibroblast activation. Both Emo-Lip and Emo-Sol significantly reduced the α-SMA^+^ cell fraction relative to TGF-β1 alone, demonstrating effective suppression of fibroblast-to-myofibroblast transition (Figure 2J,N). qPCR analysis of *Ctgf*, a downstream profibrotic mediator, revealed a parallel pattern: TGF-β1 markedly induced *Ctgf* mRNA expression, and both emodin formulations significantly suppressed this upregulation (Figure 2K). Western blot analysis was conducted to examine α-SMA protein expression, as well as the phosphorylation levels of Smad3. TGF-β1 stimulation resulted in marked upregulation of α-SMA protein levels. Both Emo-Lip and Emo-Sol significantly reduced α-SMA expression compared with the TGF-β1 group, as confirmed by densitometric quantification normalized to GAPDH (Figure 2L,M), while it inhibited the phosphorylation levels of Smad3 stimulate by TGF-β1 (Figure 2N). In general, Emo-Lip can attenuate cell proliferation, migration, oxidative stress, and myofibroblast differentiation, accompanied by downregulation of *Ctgf* mRNA expression and α-SMA protein levels activated by TGF-β1. Across most endpoints examined, free emodin exhibited slightly greater potency than the liposomal formulation under the short-term in vitro conditions, a pattern consistent with the sustained-release characteristics of liposomes that require drug liberation from the lipid bilayer prior to exerting pharmacological activity.

### 3.3. Emo-Lip Suppresses LPS-Induced M1 Polarization of RAW264.7 Macrophages

To evaluate the effect of Emo-Lip on macrophage polarization, we established an in vitro M1 polarization model using LPS-stimulated RAW264.7 macrophages. Flow cytometry was performed with CD86 and CD11b co-staining. After 24 h of LPS stimulation, a pronounced increase in the CD86^+^ population was observed in the dot plots, confirming successful M1 polarization. Both Emo-Lip and Emo-Sol treatment markedly reduced this population compared with the LPS group (Figure 3A). Flow cytometry histograms of CD86 fluorescence intensity further illustrated a rightward shift upon LPS stimulation, which was partially reversed by both Emo-Lip and Emo-Sol treatment (Figure 3B). Quantitative analysis showed that the percentage of CD86^+^ cells in the LPS group was significantly higher than that in the control group, and both emodin formulations significantly reduced this percentage (Figure 3D).

Immunofluorescence co-staining for pan-macrophage marker F4/80 and CD86 yielded consistent results. LPS stimulation strongly enhanced CD86 fluorescence intensity in F4/80^+^ macrophages. Both Emo-Lip and Emo-Sol diminished CD86 fluorescence, indicating effective suppression of M1 marker expression (Figure 3C). Quantitative analysis of F4/80^+^ cell numbers showed proliferation after LPS treatment, while CD86 relative fluorescence intensity per cell revealed a significant increase in the LPS group compared with the control, and both Emo-Lip and Emo-Sol significantly reduced this intensity relative to LPS alone (Figure 3E,F).

ELISA measurements revealed that LPS sharply elevated the secretion of IL-12 p70 and IL-6 into the culture supernatant. Both Emo-Lip and Emo-Sol significantly reduced the levels of these pro-inflammatory cytokines compared with the LPS group (Figure 3G,H). Western blot analysis was performed to examine the protein expression levels of p-p65 and total p65. LPS stimulation resulted in a marked increase in p-p65 protein levels, while total p65 expression remained stable across groups. Both Emo-Lip and Emo-Sol significantly reduced the p-p65/p65 ratio compared with the LPS group (Figure 3I,J). These data indicate that Emo-Lip effectively inhibits LPS-driven M1 polarization by interfering with NF-κB pathway activation. As in the fibroblast experiments, free emodin was modestly more potent than the liposomal form under the short-term treatment conditions.

### 3.4. Emo-Lip Attenuates Fibrous Capsule Formation and Peri-Implant Fibrosis in a Rat Silicone Implant Model

**Figure 4:**
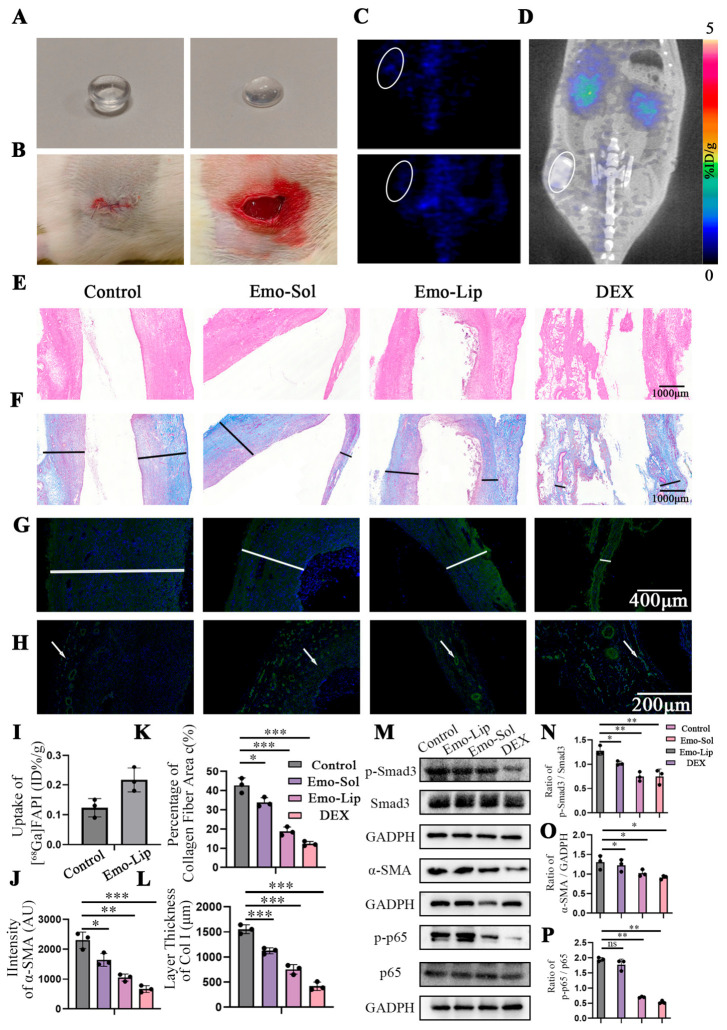
Emo-Lip attenuates fibrous capsule formation and peri-implant fibrosis in a rat silicone implant model. (**A**) Representative photograph of the silicone implant (diameter = 1 cm, thickness = 3 mm). (**B**) Representative intraoperative image showing the immediate appearance after subcutaneous implantation on the dorsum of an SD rat. The arrow indicates the implant site. (**C**) Representative FAPI-PET/CT images of the peri-implant region at 4 weeks post-implantation across treatment groups. The color scale denotes the standardized uptake value (SUV). (**D**) Quantitative analysis of FAPI uptake, expressed as the mean SUV in the peri-implant region. (**E**) Representative H&E staining images of fibrous capsule tissue at 4 weeks. Scale bar = 1000 μm. (**F**) Representative Masson’s trichrome staining of fibrous capsule tissue. Black lines indicate the average capsular thickness. Scale bar = 1000 μm. (**G**) Representative immunofluorescence images of Col I expression in the fibrous capsule. White lines indicate the average capsular thickness. Scale bar = 200 μm. (**H**) Representative immunofluorescence images of α-SMA expression in the fibrous capsule. White arrows point to the capsule. Scale bar = 400 μm. (**I**) Quantitative analysis of collagen area percentage from Masson’s trichrome staining. (**J**) Quantitative analysis of α-SMA from immunofluorescence. (**K**) Quantitative analysis of collagen area percentage from Masson’s trichrome staining. (**L**) Quantitative analysis of Col I layer thickness from immunofluorescence. (**M**) Western blot analysis of p-Smad3, total Smad3, α-SMA, p-p65 and p65 protein expression levels in fibrous capsule tissues at 4 weeks post-implantation. GAPDH was used as the loading control. (**N**) Densitometric quantification of the p-Smad3/Smad3 ratio. (**O**) Densitometric quantification of α-SMA protein expression normalized to GAPDH. (**P**) Densitometric quantification of the p-p65/p65 ratio. All data are presented as mean ± s.d. *n* = 3. * *p* < 0.05, ** *p* < 0.01, *** *p* < 0.001 versus Control group; ns, not significant.

We established a rat model of silicone implant-associated capsular contracture by subcutaneous implantation on the dorsum. Animals received local injections of saline (control), free emodin (Emo-Sol), Emo-Lip, or dexamethasone (DEX) for four weeks. Photographs of the silicone implant, dorsal subcutaneous pocket placement, and wound closure are shown in Figure 4A,B. In vivo PET/CT imaging with [^68^Ga]Ga-FAPI detected strong tracer accumulation surrounding the implants in the control group, indicating robust local fibroblast activation. Uptake was modestly reduced in the free emodin group and was further attenuated in the Emo-Lip and dexamethasone groups (Figure 4C,I), which revealed attenuated fibroblast activation protein (FAP) expression in fibrotic lesions after Emo-Lip treatment.

**Figure 5:**
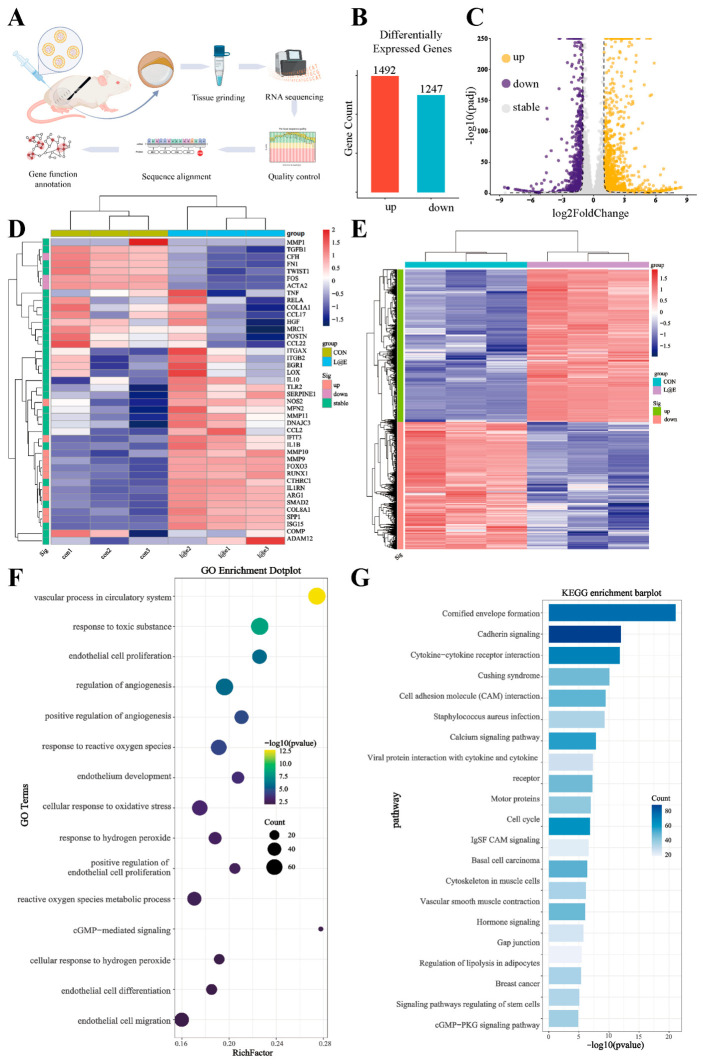
Transcriptomic analysis and in vivo Western blot validation reveal coordinated regulation of NF-κB and TGF-β/Smad pathways by Emo-Lip. (**A**) Schematic workflow of RNA-sequencing and downstream validation. (**B**) Bar chart showing the numbers of differentially expressed genes (DEGs) between the control and Emo-Lip groups. Red and blue bars represent upregulated and downregulated genes, respectively (|log_2_FC| > 1, *p* < 0.05). (**C**) Volcano plot of DEGs. Red and blue points indicate up- and downregulated genes, respectively; gray points represent non-significant genes. (**D**) Heatmap of DEG expression patterns across individual samples. Each row represents a gene, each column a sample. The color scale from blue (low) to red (high) indicates relative expression levels. (**E**) Heatmap showing the expression patterns of key DEGs related to fibrosis and inflammation across samples. (**F**) Gene Ontology (GO) enrichment analysis of DEGs, categorized into biological process (BP), cellular component (CC), and molecular function (MF). (**G**) KEGG pathway enrichment analysis of DEGs. The most significantly enriched pathways are shown, with dot size corresponding to gene count and color representing adjusted *p*-value.

At four weeks post-implantation, the fibrous capsules were harvested for analysis. H&E staining showed a dense, continuous, and thickened capsule in the control group, with loosely arranged fibroblasts and abundant extracellular matrix (Figure 5E). Free emodin reduced the overall capsule thickness and improved tissue organization. Emo-Lip produced a markedly thinner and more discontinuous capsule with reduced cellularity, approaching the effects of dexamethasone, which showed the thinnest capsule with minimal fibroblast infiltration. Masson’s trichrome staining visualized tightly packed collagen fibers in the control group, distributed in parallel or whorled arrays and occupying a large blue-stained area (42.73 ± 2.22%). Both Emo-Lip (18.79 ± 1.30%) and dexamethasone (11.60 ± 0.19%) significantly decreased the collagen-positive area. Free emodin showed moderate reduction (33.85 ± 1.42%) but remained less effective than Emo-Lip (Figure 4F,K). Col I layer thickness was quantified by immunofluorescence staining (Figure 4G). The control group exhibited a thick Col I^+^ layer measuring 1553.16 ± 98.54 μm. Free emodin significantly reduced the thickness to 1123.26 ± 64.35 μm. Emo-Lip further decreased the Col I layer to 752.58 ± 98.94 μm, approaching that of dexamethasone (416.34 ± 87.39 μm). Immunofluorescence staining detected high and widespread expression of α-SMA (2299.6 ± 158.8 AU) within the capsules of the control group (Figure 4J,L). Emo-Lip reduced the fluorescence intensity of α-SMA with effects approaching those of dexamethasone. The free emodin group showed improvement over the control but was less effective than Emo-Lip.

Safety assessment by H&E staining of major organs including liver, spleen, and kidney (SI Figure S1) revealed no structural abnormalities, inflammatory infiltrates, or necrosis in any group. Emo-Lip therefore suppresses silicone implant-induced fibrous capsule formation and collagen deposition in vivo with efficacy comparable to dexamethasone and superior to free emodin.

Western blot analysis was conducted to examine the protein expression levels of p-Smad3, total Smad3, p-p65, p65 and α-SMA in fibrous capsule lysates. The control group exhibited marked upregulation of α-SMA protein levels and substantially increased Smad3 phosphorylation, indicating activation of the TGF-β/Smad pathway. Emo-Lip treatment significantly reduced both α-SMA expression, the p-Smad3/Smad3 ratio and the p-p65/p65 ratio compared with the control group. Free emodin produced a smaller reduction in these proteins, while dexamethasone also suppressed p-Smad3 and α-SMA expression (Figure 4M–O). These data confirm that Emo-Lip exerts a coordinated inhibitory effect on the TGF-β/Smad signaling pathway at the protein level, thereby achieving effective suppression of fibroblast activation and foreign body response in vivo.

### 3.5. Transcriptomic Analysis and In Vivo Validation Reveal Coordinated Regulation of the NF-κB and TGF-β/Smad Pathways by Emo-Lip

To uncover the molecular underpinnings, we performed RNA-seq on fibrous capsule tissues comparing the control and Emo-Lip groups (Figure 5A). Differential expression analysis identified 2739 differentially expressed genes (DEGs), with 1492 upregulated and 1247 downregulated. A volcano plot showed that fibrosis- and inflammation-associated genes, including *Col1a1*, *Acta2*, *Tgfb1*, *Ccl2*, and *Mmp9*, were downregulated (Figure 5B,C). Hierarchical clustering revealed a clear separation between the transcriptomes of the two groups, reflecting a global gene expression shift induced by Emo-Lip treatment (Figure 5D). Among the DEGs, profibrotic genes (*Col1a1*, *Acta2*, *Fn1*, *Tgfb1*, *Mmp9*) and inflammatory genes (*Tnf*, *Il1b*, *Ccl2*) were suppressed. The M1 marker Nos2 declined whereas M2 markers *Mrc1* and *Arg1* rose (Figure 5E). KEGG enrichment pointed to cytokine–cytokine receptor interaction, calcium signaling, cGMP-PKG signaling, cell adhesion molecules, and vascular smooth muscle contraction (Figure 5G). GO enrichment highlighted biological processes such as oxidative stress response, angiogenesis regulation, and endothelial cell proliferation and migration (Figure 5F). The transcriptomic findings are now described with appropriate caution.

Among the differentially expressed genes, the M1-associated marker Nos2 was significantly downregulated, corroborating the anti-inflammatory effect observed at the protein level, while transcripts encoding M2-associated markers Mrc1 and Arg1 showed increased abundance, which warrants future validation at the protein and functional levels. Complementing these transcript-level findings, Western blot analysis of p-p65/p65, p-Smad3/Smad3, and α-SMA/GAPDH (Figure 4N–P) confirmed that Emo-Lip exerts a coordinated inhibitory effect on both the NF-κB and TGF-β/Smad pathways at the protein level, and broadly suppresses inflammatory and fibrotic transcriptional programs, thereby achieving coordinated control of the foreign body response.

## 4. Discussion

This study asked whether simultaneously intercepting inflammatory sensing and fibrotic execution could attenuate the peri-implant immune response and reduce fibrotic outcome. The data presented here offer experimental support for this cooperative two-node blockade strategy.

In vitro, emodin liposomes showed an independent capacity to regulate both M1 polarization in macrophages and in fibroblast activation [42,43]. Free emodin, notably, appeared more potent under these short-term conditions. This result is methodologically instructive. Liposomes are engineered for prolonged residence in vivo, not for outperforming the free drug in a static culture system over a few hours [44]. The disparity highlights a principle worth keeping in view. The disconnect between the in vitro and in vivo data underscores a fundamental point: a molecule’s intrinsic activity is meaningless without adequate delivery, and free emodin’s rapid clearance prevents it from exerting that activity at the implant site. The in vivo data reinforce the point. Across a four-week treatment course, the liposomal form suppressed fibrous capsule formation more effectively than free emodin, and immunofluorescence analysis confirmed corresponding reductions in α-SMA and Collagen I deposition. Together, these results illustrate a straightforward point: pharmacological potency in cell culture does not guarantee in vivo efficacy unless delivery is solved. Emodin possesses the necessary dual-target activity, but that activity translates into meaningful antifibrotic efficacy only after pharmacokinetic barriers are overcome [45,46,47].

Transcriptomic profiling offered a broader perspective. In treated fibrous capsule tissue, inflammatory and fibrotic genes were coordinately downregulated, and both the NF-κB and TGF-β/Smad pathways were suppressed at the protein level. This global cooperativity suggests that the mechanism may extend beyond the parallel, independent inhibition of two targets [48,49]. Molecular crosstalk between these pathways has been documented [50,51]. Simultaneously blocking both may break a self-reinforcing amplification loop rather than simply summing two separate effects. Our transcriptomic data further suggest that this cooperative effect operates at the transcriptional level to reset the immune-fibrotic balance. That said, our evidence for pathway interaction remains at the transcript and protein levels. Direct molecular interaction data are still required. While transcriptomic data suggested modulation of macrophage-related genes, the specific effect on M2 polarization was not validated by flow cytometry or cytokine profiling including IL-10 and Arg1 protein levels, requires further experimental confirmation [52]. For now, this observation is limited to the transcript level, and its functional significance awaits further experimental validation.

A comparison with existing interventions reveals the distinctive therapeutic logic of Emo-Lip. Unlike dexamethasone, which reduces fibrosis indirectly through broad immunosuppression, Emo-Lip selectively targets two defined signaling branches including NF-κB in macrophages and TGF-β/Smad in fibroblasts, thereby achieving antifibrotic efficacy without globally suppressing immune function [53]. Even among other natural product-based nanoformulations, such as those carrying resveratrol or curcumin, Emo-Lip holds a unique advantage: it simultaneously addresses the inflammatory and fibrotic arms of the foreign body response, rather than intervening in only one [54,55,56]. More fundamentally, our strategy departs from the traditional premise that fibrosis can be mitigated simply by making the implant surface less immunogenic. Instead of reducing the non-self signals emitted by the material, Emo-Lip re-educates the host response—it works intracellularly to alter how macrophages and fibroblasts interpret those same signals. These two philosophies are not mutually exclusive; one acts on the input side of the foreign body cascade, the other on the response side, and they may ultimately be combined.

Having positioned our approach among existing therapies, we next asked whether the observed antifibrotic efficacy stems from the parallel, independent inhibition of NF-κB and TGF-β/Smad in their respective cell types, or whether these pathways are functionally coupled through paracrine crosstalk that is disrupted by dual-node blockade. Our data confirm simultaneous suppression of p-p65 and p-Smad3 at the protein level, but do not establish a direct physical or functional interaction between these two pathways in the implant niche. Nevertheless, indirect coupling is plausible: NF-κB-driven cytokines from macrophages can potentiate fibroblast TGF-β/Smad signaling, while TGF-β from activated fibroblasts can reciprocally enhance macrophage NF-κB activity, forming a self-amplifying circuit. Thus, blocking both nodes simultaneously may interrupt this positive feedback loop at both the inflammatory input and the fibrotic output, potentially achieving greater efficacy than single-pathway inhibition. Definitive proof of direct NF-κB/Smad3 interaction in implant-associated fibrosis will require co-immunoprecipitation, proximity ligation assays, or genetic epistasis experiments in future studies.

These mechanistic insights, together with the safety profile of local periprosthetic delivery, which maximizes drug bioavailability while minimizing systemic exposure and avoids major organ pathology-support the translational promise of this strategy [57,58,59,60]. Looking ahead, we recognize that direct chemical modification of silicone surfaces remains technically challenging due to the inertness of medical-grade elastomers. We therefore plan to develop drug-eluting surgical meshes as an alternative delivery platform that can be combined with periprosthetic Emo-Lip injection. The present study provides a foundational rationale for such combination approaches by demonstrating that coordinated pharmacological blockade of NF-κB and TGF-β/Smad effectively suppresses the foreign body response, paving the way for more sophisticated, multi-modal anti-fibrotic regimens.

Several limitations should be acknowledged. Transcriptomic analysis uncovered multiple differentially regulated pathways, but we validated only two. The contributions of the remaining pathways are unknown. Our 4-week endpoint, while encompassing the primary phase of fibrous capsule formation and maturation as established in standard rodent implant models, cannot address the long-term durability of the anti-fibrotic effect or the potential for late-stage fibrotic remodeling after treatment cessation. Future studies will extend the observation period to 8–12 weeks and include treatment withdrawal arms to evaluate the stability of the attenuated capsule phenotype and to determine whether intermittent or sustained dosing regimens are required for long-term maintenance [61,62]. The animal model employed subcutaneous implantation in rats, and the tissue microenvironment, mechanical forces, and immunological background differ from those of clinical breast implant surgery [63]. On the formulation side, the current liposomes rely primarily on size-mediated passive accumulation. There is considerable room to improve targeting efficiency [7,64].

Future work should focus on three aspects. First, improving targeting efficiency by replacing passive EPR-based accumulation with active targeting through surface modification with cell-specific ligands and systematically comparing pan-cellular versus cell-specific inhibition to optimize the delivery platform. Second, evaluating long-term capsule stability and remodeling beyond the current endpoint. Third, transitioning to large animal models that better recapitulate the human breast implant environment. These limitations do not undermine the central strategy. They define the boundaries of our conclusions and point to directions for future investigation.

## 5. Conclusions

In summary, our data demonstrate that Emo-Lip coordinately suppresses M1 macrophage polarization and fibroblast-to-myofibroblast differentiation in vitro, and periprosthetic administration in a rat model markedly attenuated fibrous capsule formation—reducing capsule thickness, collagen deposition, and α-SMA expression to levels comparable with dexamethasone, yet without broad immunosuppression. The underlying mechanism involves the simultaneous inhibition of the TGF-β/Smad pathway in activated fibroblasts and the NF-κB pathway in pro-inflammatory macrophages. Transcriptomic and protein analyses confirmed coordinated downregulation of pro-inflammatory and pro-fibrotic gene programs, with suppression of Smad3 phosphorylation, suggesting that the dual blockade disrupts a self-amplifying positive-feedback loop rather than merely adding two independent effects. By switching the cellular interpretation of implant-derived signals rather than blunting the signals themselves, this intracellular two-node strategy offers a conceptually distinct approach to implant fibrosis and is potentially combinable with material-surface modifications that act on the input side of the foreign body cascade. Future efforts should focus on improving liposome targeting efficiency, evaluating long-term capsule stability beyond four weeks, and validating efficacy in large-animal models that more closely mimic the clinical breast implant environment. These findings establish a translational framework for using targeted dual-pathway modulation to manage implant-related fibrotic diseases.

## Data Availability

The RNA-seq data generated in this study have been deposited in the NCBI Sequence Read Archive (SRA) under BioProject ID PRJNA1467595. All other data presented in this study are available on reasonable request from the corresponding author.

## Supplementary Materials

The following supporting information can be downloaded at: https://www.mdpi.com/article/10.3390/biomedicines14071586/s1, Supplementary Materials S1: Detailed Experimental Procedures; Figure S1: Safety assessment by H&E staining of major organs including liver, spleen, and kidney.

## Abbreviations

The following abbreviations are used in this manuscript:

α-SMA	alpha-smooth muscle actin
CCK-8	Cell Counting Kit-8
Col I	collagen type I
Ctgf	connective tissue growth factor
DEGs	differentially expressed genes
DEX	dexamethasone
DLS	dynamic light scattering
EE	encapsulation efficiency
ELISA	enzyme-linked immunosorbent assay
Emo-Lip	emodin liposomes
Emo-Sol	emodin solution (free emodin)
FAPI	fibroblast activation protein inhibitor
FBR	foreign body response
GO	Gene Ontology
H&E	hematoxylin and eosin
HPLC	high performance liquid chromatography
IL-6	interleukin-6
IL-12	interleukin-12
KEGG	Kyoto Encyclopedia of Genes and Genomes
LPS	lipopolysaccharide
M1	classically activated macrophage
M2	alternatively activated macrophage
NF-κB	nuclear factor kappa B
NIH/3T3	mouse embryonic fibroblast cell line
PDI	polydispersity index
PET/CT	positron emission tomography/computed tomography
p-p65	phosphorylated p65
p-Smad3	phosphorylated Smad3
qPCR	quantitative polymerase chain reaction
RAW264.7	mouse macrophage cell line
ROS	reactive oxygen species
Smad	Mothers against decapentaplegic homolog
SUV	standardized uptake value
TEM	transmission electron microscopy
TGF-β	transforming growth factor beta
