# Supplementary material for: Coordinated Two-Node Blockade of NF-κB and TGF-β/Smad Signaling Attenuates the Foreign Body Response to Prevent Capsular Contracture
Source: Biomedicines. 2026 Jul 15;14(7):1586. doi: 10.3390/biomedicines14071586 (PMC13406193; doi:10.3390/biomedicines14071586)
Supplement: Supplementary file 1 [file biomedicines-14-01586-s001.zip › biomedicines-4386062-supplementary.pdf]

**Supplementary Materials**

## 1. Detailed Experimental Procedures

### 1.1 Characterization of Emo-Lip

Freshly prepared Emo-Lip and Emo-Lip stored at 4°C for 7 days were diluted 20-fold with PBS prior to DLS measurement (HORIBA LB-550, scattering angle 90°, laser wavelength 633 nm, 25°C, three measurements per sample). Zeta potential was measured using a Bettersize BeNano 180 Zeta at 25°C in automatic voltage mode, three measurements per sample. For TEM, samples were diluted with double-distilled water to a slightly opalescent appearance, dropped onto carbon-coated copper grids, negatively stained with 0.2% phosphotungstic acid for 1 min, air-dried under an infrared lamp, and imaged at 120 kV (HITACHI). For colloidal stability, Emo-Lip was incubated in PBS (pH 7.4) at 37°C for 14 days, with aliquots withdrawn at predetermined time points for DLS measurement. Encapsulation efficiency was determined by HPLC: a 2 mL aliquot was centrifuged to separate free drug; the pellet was dissolved in methanol, ultrasonicated for 30 min, and emodin content was quantified. EE (%) = (encapsulated drug amount / total drug input) × 100%. For cellular uptake, PKH67-labeled Emo-Lip were incubated with NIH/3T3 cells (cultured in DMEM with 10% FBS, 37°C, 5% $CO_2$) for 12 or 24 h, and uptake was quantified by flow cytometry in the FITC channel. In parallel, cells were fixed with 4% paraformaldehyde, nuclei stained with DAPI, cytoskeleton labeled with phalloidin, and imaged by confocal microscopy (Leica).

### 1.2 NIH/3T3 fibroblast fibrosis model and functional assays

NIH/3T3 fibroblasts were cultured in DMEM high-glucose medium containing 10% FBS at 37°C with 5% $CO_2$. Cells in logarithmic growth phase were stimulated with TGF-β1 (10 ng/mL). Groups: Control, TGF-β1, TGF-β1 + Emo-Sol (20 μM emodin), TGF-β1 + Emo-Lip (20 μM emodin equivalent). Cell viability was assessed by CCK-8 assay: cells in 48-well plates were treated for 24 h, CCK-8 working solution (reagent:medium = 1:10) was added, incubated at 37°C in the dark for 1 h, and absorbance at 450 nm was measured. For live/dead staining, cells were co-incubated with Calcein-AM (5 μM) and PI (10 μM) at 37°C for 15 min, rinsed, and observed under a fluorescence microscope. For Transwell migration, cells in serum-free medium were seeded into upper chambers (8 μm pore, $5 \times 10^4$ cells/well); lower chambers contained complete medium with TGF-β1 and drugs. After 24 h, inserts were fixed (4% paraformaldehyde), stained (0.1% crystal violet), and non-migrated cells wiped off. Three random fields were photographed and counted. For wound healing, confluent monolayers were scratched with a 200 μL tip, rinsed, and imaged at 0, 12, and 24 h. Wound area was measured with ImageJ; relative healing rate = $(A_0 - A_t) / A_0 \times 100\%$. For ROS detection, cells were incubated with 10 μM DCFH-DA in serum-free medium at 37°C for 30 min, rinsed, and imaged; mean fluorescence intensity per cell was quantified with ImageJ.

### 1.3 qPCR analysis of Ctgf mRNA

After NIH/3T3 cells were stimulated with TGF-β1 (10 ng/mL) and treated as described for 24 h, total RNA was extracted using TRIzol reagent according to the manufacturer's protocol. RNA concentration and purity were determined by NanoDrop 2000, and 1 μg of total RNA was reverse-transcribed into cDNA using a reverse transcription kit (Yeasen, Shanghai, China).

Real-time quantitative PCR was performed on a qPCR system with SYBR Green master mix. The primers used were:

*Ctgf*: forward 5′-GACCCAACTATGATGCGAGCC-3′, reverse 5′-TCCCACAGGTCTTAGAACAGG-3′.

*Gapdh*: forward 5′-AGGTCGGTGTGAACGGATTTG-3′, reverse 5′-TGTAGACCATGTAGTTGAGGTCA-3′.

The thermal cycling conditions were: 95 °C for 3 min, followed by 40 cycles of 95 °C for 10 s and 60 °C for 30 s. Relative mRNA expression was calculated using the $2^{-}\Delta\Delta Ct$ method with Gapdh as the internal reference. All reactions were run in triplicate.

### 1.4 RAW264.7 macrophage M1 polarization model

RAW264.7 cells were stimulated with LPS (100 ng/mL) for 24 h. Groups: Control, LPS, LPS + Emo-Sol (20 μM emodin), LPS + Emo-Lip (20 μM emodin equivalent). Cell viability was measured by CCK-8 assay at 12, 24, and 36 h as described above. For flow cytometry, cells were collected after 24 h treatment, washed, incubated with fluorochrome-conjugated anti-CD86 and anti-CD11b antibodies at 4°C for 30 min, and analyzed on a flow cytometer. $CD86^{+}$ cell percentages were quantified with FlowJo. Cytokines in supernatants were measured by ELISA (TNF-α and IL-12 kits) following the manufacturer's instructions; absorbance at 450 nm was read, and concentrations were calculated from standard curves.

### 1.5 Immunofluorescence staining

NIH/3T3 cells on confocal dishes were stimulated with TGF-β1 (10 ng/mL) and treated for 24 h, fixed with 4% paraformaldehyde (15 min, RT), permeabilized with 0.1% Triton X-100 (10 min), blocked with 1% BSA (60 min), and incubated with anti-α-SMA (1:200) and anti-Vimentin (1:200) antibodies overnight at 4°C. Alexa Fluor 488-anti-rabbit IgG (1:500) and Alexa Fluor 594-anti-mouse IgG (1:500) were applied (60 min, RT, dark), followed by DAPI (10 min). The $\alpha$-$SMA^{+}$/$Vimentin^{+}$ ratio was calculated from confocal images. RAW264.7 cells on confocal dishes were stimulated with LPS (100 ng/mL) and treated for 24 h, then processed identically. Primary antibodies: anti-F4/80 (1:200) and anti-CD86 (1:200). Secondary antibodies: Alexa Fluor 594-anti-rat IgG (1:500) and Alexa Fluor 488-anti-rabbit IgG (1:500). CD86 mean fluorescence intensity was quantified.

### 1.6 Western blot analysis

For NIH/3T3 cells (TGF-β1, 10 ng/mL, 24 h treatment), total protein was extracted with RIPA buffer (containing protease/phosphatase inhibitors) on ice, quantified by BCA assay, and 30–50 μg per lane was resolved by 10% SDS-PAGE and transferred to PVDF membranes. After blocking (5% non-fat milk, 1 h, RT), blots were probed with anti-p-Smad2/3 (1:1,000) and anti-Collagen I (1:1,000) overnight at 4°C, followed by HRP-anti-rabbit IgG (1:5,000, 1 h, RT). Signals were developed with ECL; GAPDH (1:5,000) served as loading control; ImageJ was used for quantification. For RAW264.7 cells (LPS, 100 ng/mL, 24 h), the same protocol was followed with anti-p-p65 (1:1,000) and anti-p65 (1:1,000) as primary antibodies and β-actin (1:5,000) as loading control. For in vivo fibrous capsule tissues, proteins from Sham, Model, Emo-Sol, Emo-Lip, and DEX groups were

extracted and processed identically. Primary antibodies: p-p65 (1:1,000), p65 (1:1,000), p-Smad2/3 (1:1,000), Smad2/3 (1:1,000), α-SMA (1:1,000), and GAPDH (1:5,000).

### 1.7 Rat model, imaging, histology, and safety

Male SD rats (6–8 weeks, n = 48, Hubei Biont Biotechnology; license SYXK (E) 2024-0106) were housed under SPF conditions and randomized into four groups (n = 12). Under pentobarbital anesthesia (40 mg/kg, i.p.), the dorsum was depilated, disinfected, and a 1.5 cm incision was made on each side for subcutaneous silicone disc implantation. Drugs were injected periprosthetically every other day for 4 weeks. FAPI-PET/CT was performed at week 4 post-implantation; mean SUV was calculated. After euthanasia, fibrous capsules were fixed (4% paraformaldehyde, 48 h), embedded, sectioned (5 μm), and stained with H&E and Masson's trichrome. Capsule thickness and collagen area were quantified with ImageJ. Immunofluorescence for α-SMA and Collagen I was performed as in **SI 1.6** (primary antibodies: α-SMA 1:500, Collagen I 1:200). Images were acquired with a slide scanner and analyzed with SlideViewer. Liver, spleen, and kidneys were processed for H&E staining to assess systemic toxicity (**Figure S1**).

### 1.8 Transcriptome sequencing

Fibrous capsule tissues (Control and Emo-Lip groups, n = 3 each) were homogenized in TRIzol. RNA quality was assessed by NanoDrop 2000 and Agilent 2100 Bioanalyzer (RIN ≥ 8.5). mRNA was enriched with oligo(dT) beads, fragmented to 300 bp, and reverse-transcribed. Libraries were constructed, quality-checked, and sequenced on an Illumina NovaSeq 6000 (paired-end). Clean reads were obtained by removing adaptors and low-quality reads. DEGs were identified with DESeq2 ($|\log_2FC| > 1$, adjusted $p < 0.05$). Volcano plots (ggplot2) and heatmaps (pheatmap) were generated. GO and KEGG enrichment were performed with clusterProfiler (Benjamini–Hochberg adjusted $p < 0.05$). All raw sequencing data have been deposited in the NCBI Sequence Read Archive under BioProject PRJNA1467595. The accession numbers for individual samples are as follows: Con1, SRR38741057; Con2, SRR38741056; Con3, SRR38741055; LE1, SRR38741054; LE2, SRR38741053; LE3, SRR38741052.

## 2. Supplementary Figure

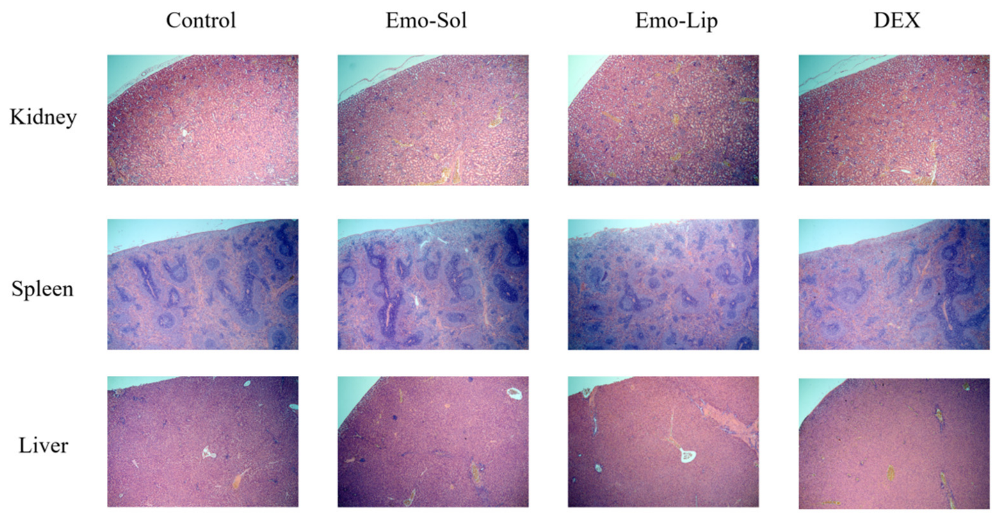


Figure S1: Safety assessment by H&E staining of major organs including liver, spleen, and kidney.
